# Shaking chills and high body temperature predict bacteremia especially among elderly patients

**DOI:** 10.1186/2193-1801-2-624

**Published:** 2013-11-21

**Authors:** Tomohiro Taniguchi, Sanefumi Tsuha, Yoshihiro Takayama, Soichi Shiiki

**Affiliations:** Division of Infectious Diseases, Okinawa Chubu Hospital, 281 Miyazato, Uruma, Okinawa, 904-2293 Japan

**Keywords:** Shaking chills, Bacteremia, Sepsis, Elderly patients

## Abstract

**Purposes:**

The difference in predictors of bacteremia between elderly and non-elderly patients is unclear despite the aging of society. The objective was to determine predictors of bacteremia among elderly patients aged 80 years and older compared to non-elderly patients aged 18 to 79 years.

**Methods:**

A referral hospital-based retrospective descriptive study from April 2012 to March 2013 in Okinawa, Japan. All enrolled patients were adults suspected of having bacterial infection who had been newly admitted into the Division of Infectious Diseases. HIV- infected patients were excluded. Exposures were a history of shaking chills, prior antibiotics use within 48 hours, vital signs, and laboratory inflammation markers on admission. Outcome was blood culture positivity.

**Results:**

Three hundred and sixty-six patients were enrolled. Median age was 78.5 (interquartile range [IQR]: 62–88). Among patients aged 18 to 79 years, shaking chills (adjusted odds ratio [AOR] 2.22, 95% confidence interval [CI]: 1.09–4.51) and previous antibiotics use (AOR 0.08, 95% CI: 0.01–0.68) were significant. However, among patients aged 80 years and older, shaking chills (AOR 3.06, 95% CI: 1.30–7.19) and body temperature above 38.5°C (AOR 2.98, 95% CI: 1.30–6.83) were significant.

**Conclusions:**

A history of shaking chills and vital signs indicating high body temperature were two findings that were useful in predicting bacteremia, especially in elderly patients aged 80 years and older. Further study is needed to assess whether the result is applicable in other regions and populations.

## Background

The incidence of sepsis and the number of sepsis-related deaths have been increasing (Martin et al. 2003). Surviving sepsis campaign guidelines have been developed in order to reduce the mortality of severe sepsis and septic shock (Dellinger et al. 2004; Dellinger et al. 2008; Dellinger et al. 2013). Early recognition and treatment of patients with sepsis is essential to prevent severe sepsis or septic shock. For this purpose, blood cultures remain the gold standard method to detect bacteremia and to identify the etiologic agents of sepsis (Aronson and Bor 1987).

The current guidelines do not clarify when blood cultures should be taken (Coburn et al. 2012). Although numerous studies have been conducted to determine the risk factors of bacteremia, the results differed among each study population (Coburn et al. 2012). One study reported that high body temperature was a predictor of bacteremia regardless of age (Bates et al. 1990). Another report in our institution demonstrated that the severity of chills was a more useful predictor than elevated temperature (Tokuda et al. 2005). Both of these results were, however, derived from relatively young patients whose average age ± standard deviation (SD) was 50±20 and 57.3±23.6, respectively.

Our hospital is located in the central area of Okinawa, Japan. Okinawa is historically well known for its genetic, environmental and social influences on longevity (R. Sadana 2013). The Division of Infectious Diseases sees all ages of adults, including those over 100. In these elderly persons it is difficult to predict bacteremia, because their symptoms of sepsis are often subtle or absent (Holloway 1986; Chassagne et al. 1996). No studies have directly examined the differences in the prediction of bacteremia between elderly and non-elderly patients in one division of a hospital.

The objective of this study was to clarify the predictors of bacteremia among elderly patients aged 80 years and older compared to those of non-elderly patients aged 18 to 79 years. Investigated predictors were a history of shaking chills, prior antibiotics use within 48 hours, vital signs, and laboratory inflammation markers. The results may prove useful for the early recognition and treatment of elderly patients with sepsis.

## Methods

### Study population

This was a hospital-based retrospective descriptive study. The study setting was Okinawa Chubu Hospital, which is located in the central area of Okinawa, a subtropical region in Japan. Approximately 39,000 patients visit the emergency department and nearly 14,000 patients are hospitalized each year. Study enrollment was from April 2012 to March 2013. All adult patients who were suspected of having bacterial infections and who were newly admitted into the Division of Infectious Diseases were enrolled in the study. The division treats all kinds of infectious diseases in adults except for those with severe lung disease, those with febrile neutropenia, those with chronic kidney disease who are on hemodialysis, or those who need endoscopic or surgical management. Exclusion criteria were 1) diagnosed with something other than a bacterial infection after culture results and 2) all human immunodeficiency virus (HIV)-infected patients. Infections that occurred after admission were not counted. All patient information on admission was collected from medical charts including vital signs such as systolic blood pressure, diastolic blood pressure, pulse rate, respiratory rate, body temperature, and laboratory results such as peripheral white blood count and C-reactive protein. Chill was defined as a perception of cold, and shaking chill was chill plus involuntary muscle tremors in order to increase body temperature. If a history of shaking chills was not recorded, we assumed that there was none. At least two sets of blood cultures which included aerobic and anaerobic bottles were routinely drawn from upper or lower limbs on admission by our interns or residents before starting antibiotics. If cultured bacteria were considered contaminants, they were classified as blood culture negatives. We set up the primary outcome as blood culture positivity. This proposal was approved by the Institutional Review Board of Okinawa Chubu Hospital.

### Statistical analysis

Patient characteristics were calculated using the chi-squared test or Fisher’s exact test for categorical variables, and Student’s *t*-test or Mann–Whitney *U* test for numerical variables after distribution analysis. A multiple logistic regression model was used to investigate risks of blood culture positivity. These were calculated by Stata software (version 12.1; StataCorp, College station, TX, USA).

## Results

Three hundred and sixty-six patients were enrolled. Mean age ± SD was 73.2±20.3; age range was 18 to 107. Table 1 shows the basic characteristics. Patients were divided into two groups: 1) aged 18 to 79 years and 2) aged 80 years and older. Sex was almost equal in the aged 18 to 79 years group, and women were dominant in the aged 80 years and older group. The blood culture positivity of each group was almost the same (23.4% vs. 25.8%). In terms of infection site, urinary tract, pulmonary, and skin & soft tissue were the top three in both groups. Twenty-one patients had two simultaneous infection sites. In terms of detected bacteria, 45 *Esherichia coli* included 3 extended-spectrum β-lactamase (ESBL) positives, 10 *Staphylococcus aureus* included 1 methicillin resistant *S. aureus* (MRSA), 6 *Klebiella pneumoniae* included 1 ESBL positive, and 9 others included *Citrobacter freundii*, *Enterococcus faecium*, *Leptospira* sp. (from Kortoff culture media), *Pseudomonas aeruginosa*, *Streptobacillus* sp., *Streptococcus parasanguinis*, *Streptococcus pneumoniae*, *Streptococcus pyogenes*, and *Vibrio alginolyticus*. Three patients had two bacterial co-infections.

**Table 1:** **Basic characteristics**

	Total	Age 18 to 79	Age above 80
	N = 366	N = 192	N = 174
Median age (Interquartile range)	78.5 (62-88)	63 (45-74)	89 (84-93)
Male sex	134 (36.6%)	89 (46.3%)	45 (25.8%)
Blood culture positivity	90 (24.5%)	45 (23.4%)	45 (25.8%)
Infection site			
Urinary tract	178 (48.6%)	99 (51.5%)	79 (45.4%)
Pulmonary	91 (24.8%)	30 (15.6%)	61 (35.0%)
Skin & soft tissue	68 (18.5%)	38 (19.7%)	30 (17.2%)
Intra-abdominal	14 (3.8%)	8 (4.1%)	6 (3.4%)
Upper respiratory	7 (1.9%)	7 (3.6%)	0
Cardiovascular	6 (1.6%)	3 (1.5%)	3 (1.7%)
Bone & joint	5 (1.3%)	4 (2.0%)	1 (0.5%)
Central nervous system	4 (1.0%)	3 (1.5%)	1 (0.5%)
Others	14 (3.8%)	8 (4.1%)	6 (3.4%)
Detected bacteria	93 (25.4%)	47 (24.4%)	46 (26.4%)
*Esherichia coli*	45	22	23
*Streptococcus dysgalactiae* subsp. *equisimilis*	11	7	4
*Staphylococcus aureus*	10	4	6
*Klebsiella pneumoniae*	6	2	4
*Enterococcus faecalis*	3	0	3
*Morganella morganii*	3	2	1
*Proteus mirabilis*	3	2	1
*Streptococcus agalactiae*	3	2	1
Others	9	6	3
Death	12 (3.2%)	3 (1.5%)	9 (5.1%)

Table 2 shows a comparison of investigated predictors between blood culture positives and negatives. Among patients aged 18 to 79, shaking chills, prior antibiotics exposure within 48 hours, and high body temperature were significant, while among patients aged 80 and older, shaking chills, tachypnea, and high fever were significant.

**Table 2:** **Investigated predictors of bacteremia within blood culture positives and negatives**

	Total			Age 18 to 79			Age above 80		
	Blood culture positives	Negatives	***p*** value	Blood culture positives	Negatives	***p*** value	Blood culture positives	Negatives	***p*** value
	N = 90	N = 276		N = 45	N = 147		N = 45	N = 129	
Male sex	28 (31.1%)	106 (38.4%)	0.212	16 (35.5%)	73 (49.6%)	0.097	12 (26.6%)	33 (25.5%)	0.886
History									
Shaking chills	47 (52.2%)	70 (25.3%)	0.000*	26 (57.7%)	51 (34.6%)	0.006*	21 (46.6%)	19 (14.7%)	0.000*
Prior antibiotics use	6 (6.6%)	64 (23.1%)	0.000*	1 (2.2%)	35 (23.8%)	0.000*	5 (11.1%)	29 (22.4%)	0.071
Vital signs (mean±SD)									
Blood pressure systolic (mmHg)	122 ± 26	119 ± 24	0.3347	118 ± 25	117 ± 24	0.8815	126 ± 26	121 ± 24	0.2523
Blood pressure diastolic (mmHg)	67 ± 13	66 ± 13	0.2877	67 ± 13	66 ± 12	0.7516	68 ± 12	65 ± 14	0.2456
Pulse rate (/min)	104 ± 20	101 ± 20	0.1877	107 ± 18	106 ± 21	0.6532	101 ± 21	95 ± 18	0.0929
Respiratory rate (/min)	24 ± 6.4	22 ± 5.0	0.0078*	22 ± 5.5	22 ± 5.0	0.4752	26 ± 6.6	23 ± 4.8	0.0033*
Body temperature (°C)	38.5 ± 1.0	37.8 ± 0.9	0.0000*	38.3 ± 1.0	38.0 ± 0.9	0.0393*	38.6 ± 1.1	37.7 ± 0.9	0.0000*
Laboratory markers (median [IQR])									
White blood count (/μl)	11000[5900]	11000[5500]	0.2259	12000[5100]	11000[5100]	0.1381	10000[4000]	10800[4800]	0.7554
C-reactive protein (mg/dl)	7.0[10.2]	5.7[10]	0.1897	10[12.2]	7.0[10.2]	0.4398	6.0[9.2]	4.1[8.5]	0.1775

*SD*, standard deviation.

*IQR*, interquartile range.

*p<0.05.

Table 3 shows the odds ratio of blood culture positivity. Among patients aged 18 to 79, the adjusted odds ratio of shaking chills was high, 2.22 (95% confidence interval [CI]: 1.09–4.51); prior antibiotics use was low, 0.08 (95% CI: 0.01–0.68). Among patients aged 80 and older, the adjusted odds ratios of shaking chills and body temperature above 38.5°C were high, 3.06 (95% CI: 1.30–7.19) and 2.98 (95% CI: 1.30–6.83) each.

**Table 3:** **Odds ratio of blood culture positivity**

	Total (N = 366)			Age 18 to 79 (N = 192)			Age above 80 (N = 174)		
	Unadjusted odds ratio (95% C.I.)	Adjusted odds ratio (95% C.I.)	***p*** value	Unadjusted odds ratio (95% C.I.)	Adjusted odds ratio (95% C.I.)	***p*** value	Unadjusted odds ratio (95% C.I.)	Adjusted odds ratio (95% C.I.)	***p*** value
Shaking chills	3.21 (1.96-5.27)	2.53 (1.50-4.28)	0.001*	2.57 (1.30-5.09)	2.22 (1.09-4.51)	0.028*	5.06 (2.36-10.84)	3.06 (1.30-7.19)	0.010*
Prior antibiotics use within 48 hours	0.23 (0.09-0.56)	0.31 (0.12-0.76)	0.011*	0.07 (0.009-0.54)	0.08 (0.01-0.68)	0.020*	0.43 (0.15-1.19)	0.66 (0.22-1.94)	0.456
Respiratory rate above 25/min	1.74 (1.01-3.00)	1.46 (0.81-2.61)	0.203	1.35 (0.57-3.18)	1.30 (0.52-3.23)	0.562	2.07 (1.00-4.27)	1.31 (0.54-2.97)	0.515
Body temperature above 38.5°C	2.64 (1.62-4.32)	1.93 (1.14-3.28)	0.014*	1.57 (0.79-3.10)	1.40 (0.68-2.88)	0.348	4.93 (2.39-10.18)	2.98 (1.30-6.83)	0.010*

*CI*, confidence interval.

*p<0.05.

The unadjusted odds ratios of leukocytosis (peripheral white blood count more than 12000/μl) or leukopenia (less than 4000/μl) among all patients, patients aged 18 to 79, and patients aged 80 and older were 1.26 (95% CI: 0.78–2.03), 1.86 (95% CI: 0.93–3.69), and 0.87 (95% CI: 0.43–1.74), respectively (not shown in the table).

We also calculated odds ratio of systemic inflammatory response syndrome (SIRS) on blood culture positivity. The unadjusted odds ratio among all patients, patients aged 18 to 79, and patients aged 80 and older were 1.78 (95% CI: 1.02–3.07), 1.93 (95% CI 0.88–4.27), and 1.64 (95% CI: 0.76–3.51), respectively (not shown in the table).

## Discussion

Our study had three main findings. First, a history of shaking chills was the highest predictor of blood culture positivity regardless of age. Second, prior antibiotics use within 48 hours was the lowest effect only in patients aged 18 to 79 years, not in patients aged 80 years and older. Third, body temperature above 38.5°C was a high effect only in patients aged 80 years and older.

Shaking chills or shivering is defined as a perception of cold and involuntary muscle tremors that persist for several minutes and are accompanied by a rise in body temperature (Van Dissel et al. 2005). A history of shaking chills was highly associated with bacteremia (Coburn et al. 2012; Van Dissel et al. 1998; Bates et al. 1990; Stryjewski et al. 2009; Leibovici et al. 1991; Van Dissel et al. 2005), and it was also confirmed among patients aged 65 years and older (Fontanarosa et al. 1992).

In our research, shaking chills had the greatest effect on blood culture positivity (adjusted odds ratio [OR] 2.53, 95% CI: 1.50–4.28) –this is consistent with previous reports. The effect was a little higher in patients 80 years and older (adjusted OR 3.06, 95% CI: 1.30–7.19) than patients aged 18 to 79 years (adjusted OR 2.22, 95% CI: 1.09–4.51).

We assumed that infections in elderly patients were likely to become exacerbated, because their presentation of bacteremia was often atypical or nonspecific (Pfitzenmeyer et al. 1995) and as a result, the people around them were delayed in noticing a change. Elderly persons were more susceptible to temperature stress because of impaired behavioral, shivering, vasomotor, and sudomotor responses (Norman et al. 1985). Many patients who had mental disorders were unable to complain of chills and were thus unable to increase their body temperature. Immune host defenses were not activated in the absence of fever (Pien et al. 2010). Finally, shaking chills occur when the burden of bacteremia is high.

The severity of chills graded on an ordinal scale such as mild, moderate, and shaking chills would be more useful than binominal scale such as shaking chills positive or negative (Tokuda et al. 2005). However, the self-reporting of the severity of chills in patients with dementia was not reliable. In addition, it was frequently difficult to evaluate bacteremia of such patients. Shaking chills were easily recognized objectively by people around them.

A history of shaking chills is more than just useful information for predicting bacteremia in adults. Even among patients with a cognitive disorder, it is a simple and objective finding.

Prior use of antibiotics lower the rate of blood culture positivity (Aronson and Bor 1987). Although some reports found no association between previous antibiotics usage and bacteremia (Tokuda et al. 2005; Bates et al. 1990; de Jager et al. 2010), blood cultures should be drawn before antibiotics start.

Our study demonstrated that prior antibiotics use within 48 hours was a significantly low effect on blood culture positivity only among patients aged 18 to 79 years. Because types of prior antibiotics were not evaluated, however, it was uncertain whether the selected antibiotics were effective against etiologic agents. However, as discussed above, elderly patients were more likely to have a high burden of bacteremia. Prior antibiotics use in such patients would not be sufficient to diminish bacteremia.

This research reconfirms that blood cultures should be taken before antibiotics treatment especially among patients aged 18 to 79 years. In patients aged 80 years and older, blood cultures should be drawn without delay, even after antibiotics have already been started.

According to the definition of SIRS, tachycardia, tachypnea, high or low body temperature, leukocytosis or leukopenia are predictors of sepsis (American College of Chest Physicians/Society of Critical Care Medicine Consensus Conference 1992). SIRS was a sensitive, but not specific indicator of bacteremia (Coburn et al. 2012). The positive predictive value of SIRS for predicting bacteremia was only 7% (Jones and Lowes 1996).

In this study, SIRS might be useful for all patients (unadjusted OR 1.78, 95% CI: 1.02–3.07), but not useful in each age group. Among all vital signs, high body temperature (above 38.5°C) was only a high predictor in elderly patients aged 80 years and older (adjusted OR 2.98, 95% CI: 1.30–6.83), though antipyretics use was not described. Tachypnea (above 25/min) may be a predictor for them (unadjusted OR 2.07, 95% CI: 1.00-4.27), although it was not significant after adjustment. Within patients aged 18 to 79 years, there was no indicator of vital signs that affected blood culture positivity despite our calculations of several cut-off values.

Although some reports have suggested that high body temperature was a predictor of bacteremia (Bates et al. 1990), fever was an inaccurate predictor of bacteremia (Coburn et al. 2012). However, research performed at a geriatric hospital showed that fever was a predictor of bacteremia (Pfitzenmeyer et al. 1995). The average age was 83.8±6.5 years, and the best relative risk was noted at a cut-off value of 38.5°C. Our research findings were consistent with this.

High body temperature may not be a precise indicator of bacteremia, but it is a valuable finding especially among patients aged 80 years and older. Tachypnea may be a notable finding for them.

Although laboratory inflammation markers such as peripheral white blood count and C-reactive protein were predictors of bacteremia in previous research (de Jager et al. 2010), they were useless in our study for both age groups. Nevertheless, we calculated the odds ratio of leukocytosis or leukopenia according to SIRS.

This study had several limitations. First, the study was set in a referral hospital in Okinawa, a subtropical region of Japan. Most patients were Japanese. There is endemic leptospirosis in this area, but no malaria which frequently causes shaking chills. Second, a selection bias in the Division of Infectious Diseases existed. We did not attend to patients with intra-abdominal infection such as ascending cholangitis or patients with chronic kidney disease on hemodialysis. Third, we were not sure of the timing and volume of blood cultures, despite the fact that our attending doctors educated residents to take blood cultures as soon as shaking chills took place, and to obtain at least 10 ml in each set.

## Conclusions

The prediction of bacteremia in elderly patients is always challenging for clinicians because of their subtle or absent symptoms. However, a history of shaking chills, vital signs of high body temperature, and possibly tachypnea are significant clues. Confirmation of these findings would lead to the early recognition and treatment of sepsis. Even if antibiotics have been started improperly without obtaining blood cultures among elderly patients, blood cultures should be drawn because prior antibiotics use among them didn’t affect blood culture positivity in our research. Further study is needed to assess whether our result is applicable in other regions and populations.
